# Nomogram Based on Monocyte-to-Lymphocyte Ratio to Predict Survival of Unresectable Esophageal Squamous Cell Carcinoma Who Receive First-Line PD-1/PD-L1 Inhibitors Combined with Chemotherapy

**DOI:** 10.3390/curroncol29110702

**Published:** 2022-11-18

**Authors:** Xiaolu Ma, Yongfeng Ding, Jiong Qian, Mingyu Wan, Ning Li, Chenyu Mao, Cheng Xiao, Haiping Jiang, Yulong Zheng, Luntao Wu, Xiaoyu Chen, Nong Xu

**Affiliations:** Department of Medical Oncology, The First Affiliated Hospital, School of Medicine, Zhejiang University, Hangzhou 310003, China

**Keywords:** esophageal squamous cell carcinoma, PD-1/PD-L1, immunotherapy, monocyte-to-lymphocyte ratio, nomogram

## Abstract

Background. Chemoimmunotherapy has become the first-line treatment for unresectable esophageal squamous cell carcinoma (ESCC). Still, reliable biomarkers to identify patients who could benefit from this combined therapy remain uncertain. This study focused on elucidating the predictive significance of the monocyte-to-lymphocyte ratio (MLR) and establishing the prognostic nomogram for unresectable ESCC treated with chemoimmunotherapy. Methods. Data of clinical features, peripheral blood parameters, and treatment records were collected in unresectable ESCC patients who received PD-1/PD-L1 inhibitors plus chemotherapy as the first-line treatment from September 2017 to August 2021. The nomogram based on MLR and clinical parameters for predicting the overall survival (OS) was developed and validated. Results. Out of 81 patients enrolled, patients with a lower MLR had significantly longer progression-free survival (PFS) and OS than patients with a higher pretreatment MLR (*p* = 0.0067; *p* = 0.00069). The OS nomogram integrating MLR, performance status (PS) score, and body mass index (BMI) achieved a C-index of 0.770 (95%CI 0.645–0.896). The area under the ROC curve (AUC) value of the nomogram predicting 12-, 18-, and 24-month OS rates were 0.855, 0.792, and 0.744, respectively, which were higher than the clinical TNM staging system or the MLR. Stratified by the nomogram-generated scores, three risk groups (low, moderate, and high) in survival curves manifested a distinct difference (*p* < 0.0001). Conclusion. MLR emerged as an independent predictive factor for PFS and OS in treatment-naive unresectable ESCC patients treated with chemoimmunotherapy. The constructed nomogram of MLR and clinical parameters was a reliable model for prognostic estimation.

## 1. Introduction

Esophageal cancer is the seventh most common cancer globally and the sixth leading cause of cancer-related death [1]. Compared to European and American countries, where adenocarcinoma is the most common type, approximately 90% of esophageal cancer in China is esophageal squamous cell carcinoma (ESCC) [2]. The lack of typical clinical symptoms and sensitive tumor biomarkers makes early detection of ESCC a challenge. Patients are often diagnosed at an advanced stage without a chance of radical surgery; such patients are evaluated as unresectable. The unresectable patients are further divided into two categories: locally advanced and metastatic, which differ in the conventional therapeutic strategies [3]. Concurrent chemoradiotherapy has been the wildly accepted approach for locally advanced ESCC. The mainstay of first-line treatment for metastatic or recurrent ESCC is platinum-based systemic chemotherapy [4,5]. However, the five-year overall survival (OS) rate for unresectable ESCC, even when classified as locally advanced, is around 20% [6].

The emergence of PD-1/PD-L1 immune checkpoint inhibitors (ICIs) has advanced the therapeutic landscapes of various cancers, particularly ESCC. Phase III trials have revealed that anti-PD-1 inhibitor monotherapy, compared to conventional palliative chemotherapy, could provide better OS for advanced ESCC in the second- or later-line setting [7,8]. The following randomized controlled trials such as KEYNOTE-590 [9], CheckMate 648 [10], ESCORT-1st [11], and ORIENT-15 [12] have investigated the efficacy of PD-1 inhibitors combined with chemotherapy in the first-line setting, and consistently demonstrated an outstanding survival optimization for advanced ESCC [13]. Additionally, the results of JUPITER-06 were also published recently [14], suggesting that adding an anti-PD-1 agent to chemotherapy has a superior OS vs. chemotherapy alone in the first-line setting (median OS 17.0 vs. 11.0 months, HR 0.58, *p* = 0.0004). Based on these findings, the anti-PD-1 agent combined with chemotherapy has been recommended as the standard first-line therapeutic regimen for advanced ESCC.

Despite the PD-1 pathway blockade showing durable antitumor capacity in partial ESCC patients, a subset of patients remained resistant to this immunotherapy. The PD-L1 expression detected by immunohistochemistry was closely correlated with the prediction of efficacy of the PD-1/PD-L1 blockade [15]. Several studies have found that PD-L1-negative patients responded to PD-1/PD-L1 inhibitors and a population with its positive expression exhibited no superior survival benefit from the same treatment [8,16]. Moreover, given the variety of assay methods and tumor heterogeneity, it is difficult to compare and standardize [17]. Meanwhile, other tumor-specific predictive candidates such as tumor mutational burden (TMB), circulating tumor DNA (ctDNA), and gut microbiota have potential clinical practice limitations [18,19]. High expenditure and time consumption of these detections necessitate an accessible and cost-effective biomarker to predict the benefit of the PD-1/PD-L1 pathway inhibitors.

Numerous evidence has indicated that inflammation is related to the carcinogenesis, proliferation, infiltration, and metastasis of the tumor [20]. The neutrophil-to-lymphocyte ratio (NLR) and the monocyte-to-lymphocyte ratio (MLR) are recognized as the hallmarks of systemic inflammatory status that can reflect the prognosis of ICIs-received solid tumors: non-small-cell lung cancer (NSCLC) [21], melanoma [22], and gastric cancer (GC) [23,24]. Recent research has looked into the prognostic value of inflammatory indicators in ESCC patients who have received the anti-PD-1 immunotherapy [25,26,27]. In their studies, patients in the second or later lines make up a large proportion. Since immunotherapy combined with chemotherapy has been introduced as a first-line treatment for ESCC, this study sought to identify an optimal inflammatory biomarker for predicting the prognosis of previously untreated ESCC patients who received PD-1/PD-L1 ICIs plus chemotherapy.

This study assessed whether NLR or MLR could be a reliable predictor of survival for treatment-naive locally advanced or metastatic ESCC patients who had received PD-1/PD-L1 antibodies combined with chemotherapy as the first-line treatment. Moreover, to identify the high-risk population and optimize the clinical therapeutic paradigm, it is critical to establish a predictive nomogram by incorporating simple clinical features and accessible serum biomarkers into the individual prognostic analysis.

## 2. Materials and Methods

### 2.1. Study Population

**Figure 1:**
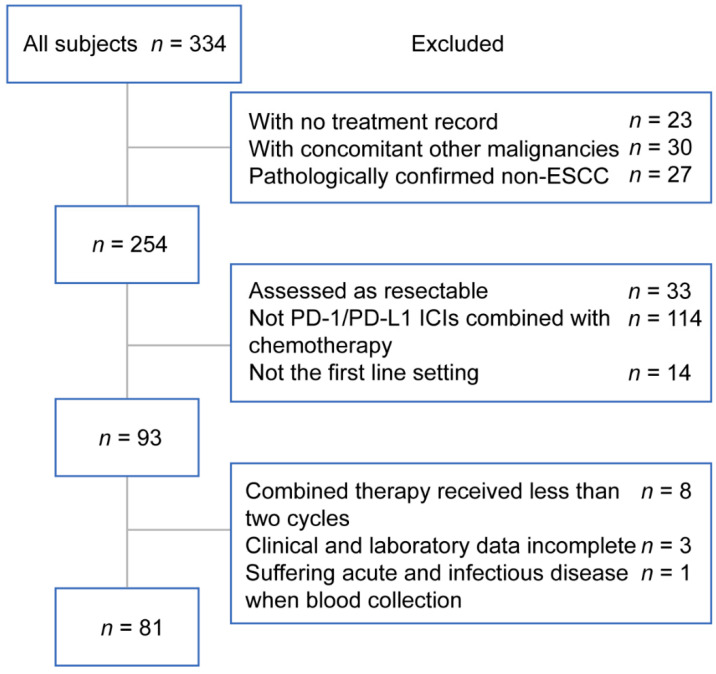
The flowchart presented the screening process for eligible patients. Abbreviations: PD-1, programmed death-1; PD-L1, programmed death ligand-1; ICIs, immune checkpoint inhibitors.

Data was collected retrospectively from 334 patients diagnosed as ESCC in the Medical Oncology Department of the First Affiliated Hospital of Zhejiang University School of Medicine between September 2017 and August 2021. At last, 81 patients met the requirements for the final analysis (Figure 1). The inclusion criteria were as follows: (1) pathologically diagnosed as esophageal squamous cell carcinoma; (2) assessed as unresectable and precluded definitive chemoradiation; (3) accepted PD-1/PD-L1 ICIs combined with chemotherapy as the first-line treatment for two cycles at least; (4) complete baseline clinical and laboratory data available; (5) radiological examinations before and after the treatments available. The exclusion criteria were as follows: (1) with concomitant other malignancies; (2) had dysfunction of vital organs; (3) acute and infectious disease suffered when blood collection; (4) with active autoimmune disorder or severe immune deficiency. All patients were staged by the eighth version of American Joint Committee on Cancer (AJCC) tumor-node-metastasis (TNM) staging system. During this period, 60 patients diagnosed as ESCC who were assessed as unresectable and received chemotherapy as the first-line treatment were also included for a further control. These patients received at least two cycles of first-line chemotherapy and were never treated with immunotherapy. The study adhered to the principles of the Declaration of Helsinki and this study protocol was approved by the ethics committee of the First Affiliated Hospital of Zhejiang University School of Medicine. Individual consent was needless for this retrospective analysis.

### 2.2. Data Collection

Besides the process of treatment, clinical data of patients including gender, age, smoking history, alcohol status, tumor location, site of metastasis, TNM stage, Eastern Cooperative Oncology Group (ECOG) performance status (PS) score, and body mass index (BMI) was extracted from the electronic medical records. Meanwhile, peripheral blood parameters were collected within four weeks before the first-line combined treatment initiation. The baseline MLR was defined as the ratio of monocyte count to lymphocyte count. The baseline NLR was defined as the neutrophil count divided by the lymphocyte count. Time-dependent receiver operating characteristic (timeROC) analysis for predicting 12-month OS determined the appropriate cut-off value of MLR was 0.40 and 3.67 for NLR.

### 2.3. Treatments

A total of 81 patients were all treated with anti-PD-1/PD-L1 antibodies combined with chemotherapy in the first-line setting. The varieties of anti-PD-1 antibodies were as follows: nivolumab, toripalimab, pembrolizumab, sintilimab, tislelizumab, and camrelizumab. Moreover, five patients took sugemalimab (a PD-L1-targeting antibody) as another choice. The chemotherapy regimens contained: cisplatin/carboplatin plus albumin-bound paclitaxel (nab-PTX), cisplatin/carboplatin plus paclitaxel (PTX), cisplatin/carboplatin plus fluorouracil (5-FU), and others. The combined regimen and dose were based on the patient’s actual condition and preference.

### 2.4. Assessment

According to the Response Evaluation Criteria In Solid Tumors (RECIST Version 1.1), the response of patients to the combined regimen included: complete response (CR), partial response (PR), stable disease (SD), and progressive disease (PD). Objective remission rate (ORR) was defined as CR plus PR, and disease control rate (DCR) was defined as CR plus PR plus SD. The primary endpoints were progression-free survival (PFS) and overall survival (OS). PFS was defined from the date of the first-line combined treatment initiation to the first radiographic evaluation of disease progression or last follow up. OS was defined as the time between starting the first-line combined treatment and death from any cause or last follow up. The follow-up time was ended on 5 November 2021.

### 2.5. Statistical Analysis

IBM SPSS Statistics version 26.0 software and R project ×64 version 4.1.1 were used to process clinical data and graph plotting. R package “survivalROC” was utilized to establish timeROC curves. The chi-squared and the Fisher’s exact tests were used for comparison among categorical variables. Survival curves of PFS and OS were drawn by the Kaplan–Meier method. The log-rank test was used to estimate the statistical differences between groups in term of PFS and OS. Univariate and multivariate Cox regression analyses were performed the Cox proportional hazard regression model. Factors which *p* < 0.10 in univariate analyses would be selected into multivariate analyses. Hazard ratio (HR) and 95% confidential interval (CI) were utilized to calculate relative risk. R package “rms” was used to establish the nomogram. Additionally, its accuracy was estimated by Harrell’s concordance index (C-index). Calibration curves by 1000 bootstrap resamples were conducted for internal validation. TimeROC curves were used to compare the prediction efficacy among the nomogram, the TNM staging system and the MLR risk-rating. Forest plot was created with the “forestplot” package of R. A two-sided *p*-value < 0.05 was recognized as statistical significance.

## 3. Results

### 3.1. Patients Characteristics

A total of 81 patients met the specified criteria and were enrolled in the further analysis. The clinical characteristics of them are summarized in Table 1. Of these, 74 (91.36%) were male and 7 (8.64%) were female, with an average age of 62.5 ± 8.6 years (range 44–88 years). The majority of patients (77.78%) had a history of smoking. Meanwhile, there were 58 (71.6%) patients with the habit of drinking. A total of 19 (23.46%) patients had a BMI level ≤18.5 kg/m^2^. Most patients (76.54%) were diagnosed as stage IV, and 19 (23.46%) were diagnosed as stage III. There were 20 (24.69%) patients with tumor distant metastasis and 61 (75.31%) without tumor distant metastasis. The PS score of all patients was 0 (61.73%) or 1 (38.27%). A total of 20 (24.69%) cases of ESCC patients were with a low level of hemoglobin (≤120 g/L). Of the total of 81 ESCC patients, 4 (4.94%) patients achieved CR, 53 (65.43%) patients achieved PR, 19 (23.46%) patients remained in the stable condition, and 5 (6.17%) patients had the disease progression. To sum up, the best objective response rate was 70.4% (57/81), and the best disease control rate was 93.8% (76/81). The average follow-up time of the total enrolled patients was 14.5 ± 9.5 months (range 2.1–45.1 months).

Based on the 12-month OS, the optimal cut-off values of NLR and MLR were 3.67 and 0.40, respectively. To further explore the prognostic value of NLR and MLR, the enrolled patients were divided into the NLR ≤ 3.67 group (*n* = 50), the NLR > 3.67 group (*n* = 31), the MLR ≤ 0.40 group (*n* = 45), and the MLR > 0.40 group (*n* = 36). As is shown in Table 2, the NLR > 3.67 group was significantly related to the male sex (*p* = 0.040) and a ≤120 g/L hemoglobin level (*p* = 0.021). The MLR > 0.40 group was significantly associated with the male sex (*p* = 0.015) and a distant metastasis (*p* = 0.033), especially liver metastasis (*p* = 0.041). Beyond that, a more advanced TNM stage (*p* = 0.019) and poorer performance status (*p* = 0.004) were also significantly associated with an MLR > 0.40.

### 3.2. Prognostic Analysis Based on NLR or MLR

**Figure 2:**
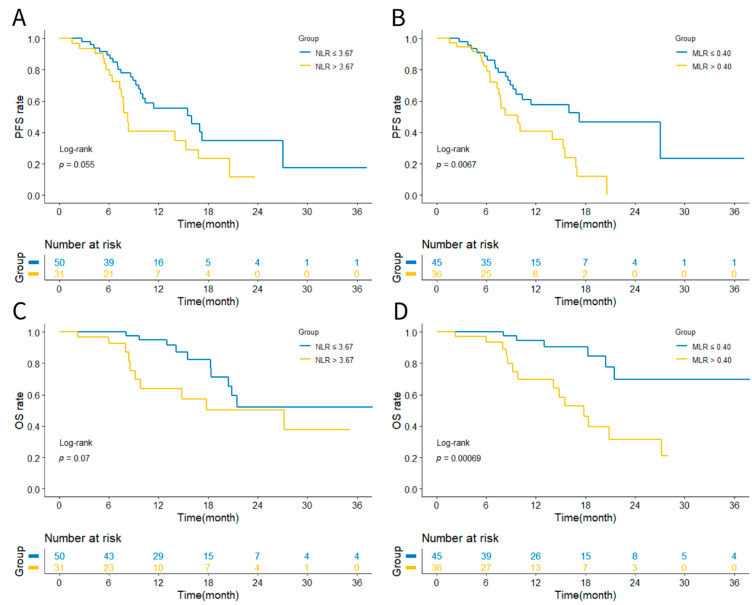
Kaplan–Meier survival curves of NLR and MLR. (**A**) PFS curves of NLR. (**B**) PFS curves of MLR. (**C**) OS curves of NLR. (**D**) OS curves of MLR. Abbreviations: NLR, neutrophil-to-lymphocyte ratio; MLR, monocyte-to-lymphocyte ratio; PFS, progression free survival; OS overall survival.

Through the Kaplan–Meier analysis and log-rank test, we compared the outcomes of patients allocated to the low level and high level of the NLR/MLR groups in terms of PFS and OS. As shown in Figure 2, the PFS rate was considered comparable between the NLR ≤ 3.67 and NLR > 3.67 (HR 0.557, 95%CI 0.286–1.083 *p* = 0.055). NLR ≤ 3.67 was not significantly associated with prolonged OS (HR 0.455, 95%CI 0.177–1.172 *p* = 0.07). However, the MLR > 0.40 had shorter PFS than the MLR ≤ 0.40 (HR 2.252, 95%CI 1.180–4.298 *p* = 0.0067). Meanwhile, there was a significant difference in OS between the MLR > 0.40 and MLR ≤ 0.40 (HR 4.495, 95%CI 1.784–11.326 *p* = 0.00069). Therefore, we proposed that the MLR could represent as the sensitive inflammatory biomarker for predicting the prognosis of previously untreated ESCC who received PD-1/PD-L1 ICIs combined with chemotherapy.

To further confirm the prognostic value of the MLR, we collected clinical variables and survival data from 60 cases of patients with unresectable ESCC during the same period. These patients all received at least two cycles of chemotherapy in the first-line setting and were never treated with immunotherapy. This treatment cohort was similar to the immunotherapy plus chemotherapy cohort in clinical characteristics such as sex, age, BMI, tumor location, metastasis condition, TNM stage, and PS score (Table S1). Later, we used the MLR value of 0.40 as a cut-off value to divide this cohort into the MLR > 0.40 group and the MLR ≤ 0.40 group (Figure S1). The Kaplan–Meier curves also showed the significant difference of the MLR > 0.40 group vs. the MLR ≤ 0.40 group in term of OS (HR 2.054, 95%CI 1.081–3.905 *p* = 0.014). The forest plot (Figure S2) exhibited patients with the lower MLR were associated with a decreased risk for death, whether in the immunotherapy plus chemotherapy cohort (HR 0.219, 95%CI 0.084–0.573) or the chemotherapy cohort (HR 0.470, 95%CI 0.254–0.869). To summarize, the MLR was a reliable biomarker for predicting the prognosis in ESCC, and it seems to perform better in the treatment with PD-1/PD-L1 ICIs plus chemotherapy.

### 3.3. Univariate and Multivariate Cox Proportional Analyses of Prognostic Factors

Univariate and multivariate survival analyses were conducted to further evaluate the prognostic value of the MLR and search for other potential affecting factors (Table 3). Univariate analyses revealed that BMI (≤18.5 kg/m^2^ vs. >18.5 kg/m^2^, *p* = 0.066), TNM stage (III vs. IV, *p* = 0.080), and the MLR (≤0.40 vs. >0.40, *p* = 0.008) were associated with PFS. Then, they were incorporated into the multivariate Cox proportional hazard model for further analyses. Multivariate analyses suggested that the MLR > 0.40 (HR 2.254, 95%CI 1.188–4.276, *p* = 0.013) was a significant prognostic biomarker for predicting inferior PFS. In terms of OS, univariate analyses revealed that BMI (≤18.5 kg/m^2^ vs. >18.5 kg/m^2^, *p* = 0.036), PS score (0 vs. 1, *p* = 0.046), and the MLR (≤0.40 vs. >0.40, *p* = 0.002) were strongly related with OS. Multivariate analyses calculated that BMI ≤ 18.5kg/m^2^ (HR 3.093, 95%CI 1.223–7.821, *p* = 0.017) and the MLR > 0.40 (HR 4.524, 95%CI 1.681–12.175, *p* = 0.003) were significant and independent prognostic parameters for predicting worse OS.

### 3.4. Construction and Validation of the OS Nomogram

**Figure 3:**
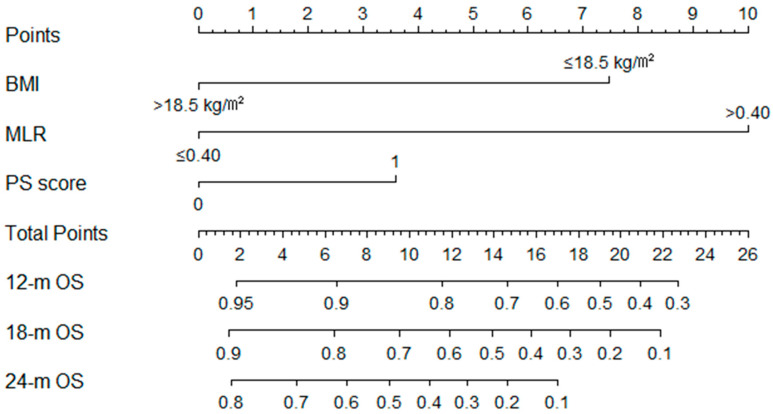
Nomogram for OS predicted the 12-, 18-, and 24-month survival probabilities. Abbreviations: BMI, body mass index; MLR, monocyte-to-lymphocyte ratio; PS score, performance status score; OS, overall survival.

**Figure 4:**
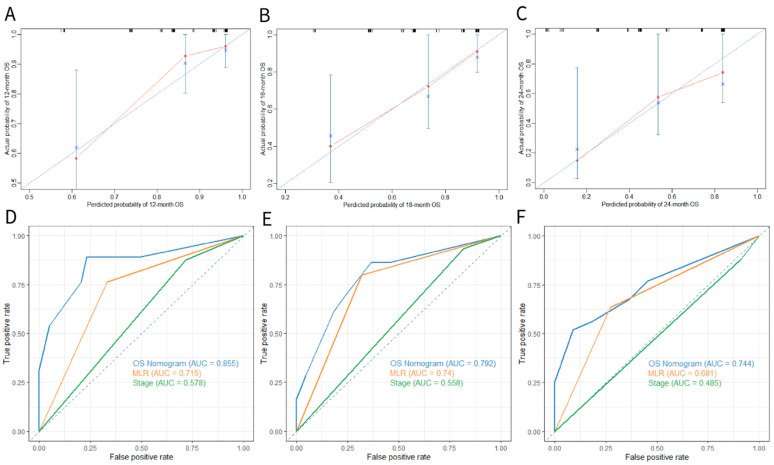
(**A**–**C**) The calibrate curves of 12-, 18-, and 24-month OS presented the consistency of predicted probabilities and actual observations. (**D**–**F**) The time-dependent ROC curves of 12-, 18-, and 24-month OS predicted by the OS nomogram, MLR and the TNM staging system respectively. Abbreviations: OS, overall survival; MLR, monocyte-to-lymphocyte ratio; TNM, tumor-node-metastasis; AUC, area under ROC curve.

To predict the survival probabilities at 12-, 18-, and 24-month time points, we established the nomogram that integrated BMI, PS score, and MLR three parameters (Figure 3). The C-index of BMI was 0.648 (95%CI 0.526–0.769), the C-index of the PS score was 0.635 (95%CI 0.519–0.750), and the C-index of the MLR was 0.701 (95%CI 0.600–0.802). However, the C-index of the nomogram was 0.770 (95%CI 0.645–0.896), demonstrating a good predictive power for overall survival. Additionally, the calibrate curves for internal validation of the nomogram illustrated good consistency between predicted possibilities and actual observations (Figure 4A–C). Moreover, the AUC of 12-, 18-, and 24-month ROC curves showed that the nomogram provided a higher value of AUC compared to that of the eighth TNM staging system or the MLR (Figure 4D–F).

### 3.5. Application of the Nomogram for Risk Stratification

**Figure 5:**
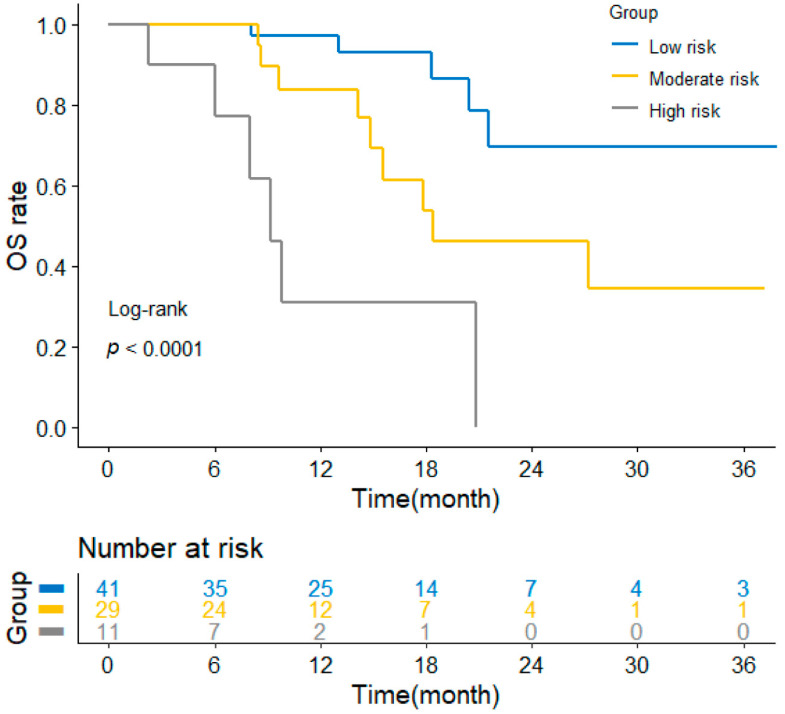
Kaplan–Meier curves of OS for ESCC patients in low-, moderate-, and high-risk groups stratified by the nomogram-generated scores. Abbreviations: OS, overall survival; ESCC, esophageal squamous cell carcinoma.

To further assess the feasibility of this constructed model, all patients were given a nomogram-generated score after that they were classified into three groups: low-risk group (score < 10, *n* = 41), moderate-risk group (10 ≤ score < 15, *n* = 29), and high-risk group (score ≥ 15, *n* = 11). The Kaplan–Meier curves (Figure 5) displayed that the low-risk group had the best OS, and the high-risk group had significantly the worst OS (*p* < 0.0001). Moreover, the median OS of three groups were not applicable (NA), 18.4 months, and 9.2 months, respectively. In summary, the OS nomogram incorporated BMI, PS score, and the MLR that had a better performance in predicting the survival outcome of previously untreated ESCC patients who received PD-1/PD-L1 ICIs plus chemotherapy and contributed to guiding clinical practice.

## 4. Discussion

This retrospective study found that the monocyte-to-lymphocyte ratio (MLR) is compatible as the reliable prognostic factor for previously untreated locally advanced or metastatic esophageal squamous cell carcinoma (ESCC). The MLR-elevated group had a larger proportion of males, a higher incidence of distant metastasis, a more advanced TNM stage, and worse performance status. Patients with an elevated MLR at baseline manifested worse PFS (*p* = 0.0067) and especially a shorter OS (*p* = 0.00069). The MLR was an independent prognostic factor for both PFS and OS in multivariate survival analyses. The constructed prognostic nomogram incorporating the MLR and other clinical variables has a better predictive accuracy of OS in unresectable locally advanced or metastatic ESCC patients.

The MLR, an inflammatory biomarker, is a significant prognostic factor in various types of malignant solid tumors treated with traditional treatments, such as colorectal cancer (CRC), nasopharyngeal carcinoma, and gastroesophageal cancer [28,29]. For instance, Jiang et al. conducted a meta-analysis of patients with esophageal cancer who underwent surgery, chemotherapy, or radiotherapy and found that an elevated MLR at baseline was an unfavorable prognostic element for OS [29]. Since the approval of pembrolizumab in 2014 for the treatment of advanced melanoma, PD-1/PD-L1 ICIs have revolutionized treatment modes ranging from Hodgkin lymphoma to advanced endometrial carcinoma [30]. Current findings validated the predictive value of the MLR in ICIs-treated patients. Chen et al. reported that a lower baseline and six-week MLR were associated with superior PFS and OS in patients with gastric cancer (GC) who received anti-PD-1/PD-L1 therapy [23]. Fan et al. demonstrated that an elevated pretreatment MLR was correlated with shorter PFS and OS for patients with advanced gastrointestinal cancer who accepted anti-PD-1 treatment [24]. Zhu et al. suggested that a high MLR was related to a short time to progression (TTP) in ICIs-received hepatocellular carcinoma (HCC) patients [31]. This study showed that treatment-naive ESCC patients with an elevated MLR treated with immunotherapy combined with chemotherapy had inferior PFS and OS. Despite a retrospective study, the MLR at baseline was unrelated to tumor response and PFS in ESCC patients who received anti-PD-1 therapy [32]. The difference compared with our work was that their study enrolled a big proportion of pretreated patients with at least one prior therapy. In clinical practice, immunotherapy combined with chemotherapy is preferred for newly diagnosed unresectable ESCC. To the best of our knowledge, this is the first study to report the prognostic significance of MLR in unresectable ESCC with ICIs-based treatment in the first-line setting.

The mechanisms by which an increased MLR is associated with the inferior outcomes of patients with ICIs-based treatment are not entirely defined. The following are the probable explanations for the correlations. First, lymphocytes infiltrating the tumor microenvironment played a pivotal role in immune surveillance and antitumor cytotoxicity [33]. However, the decline of absolute lymphocyte count in peripheral blood was frequently detected in advanced solid tumors [34,35]. It caused the insufficient resupply of tumor-infiltrating lymphocytes (TILs) from blood circulation, promoting tumor cell proliferation and invasion [36,37,38]. Since PD-1/PD-L1 inhibitors work by blocking the PD-1/PD-L1 pathway to reactivate T lymphocyte-mediated immune response [30,39], the low number of circulating lymphocytes indicated limited immunological activation. Meanwhile, studies reported tumor and stromal cells in the tumor microenvironment release chemokines (for instance, CCL2 and CCL5) and cytokines (such as CSF-1 and VEGF), recruiting circulating monocytes, resulting in tumor-associated macrophages (TAMs) [40,41]. Though “M1” macrophages possess the competence of tumor killing and pathogen clearance, TAMs always have an “M2” phenotype that fosters tumorigenesis, angiogenesis, and immune evasion [42,43]. Studies also found that TAMs could impair the activity of TILs and further aggravate immune tolerance [44]. Additionally, experimental evidence showed the depletion of TAMs could restore tumor surveillance by CD8 T cells and improve the efficacy of anti-PD-1 treatment [45]. As a result, the elevated MLR might be closely related to an immunosuppressive microenvironment that is unfavorable to ICIs-based treatment.

According to the results from multivariate analyses, lower body mass index (BMI ≤ 18.5 kg/m^2^) was associated with poor OS, which has been reported in other ICIs-treated malignant tumors [24,46,47]. Patients with advanced malignant cancers are usually characterized by rapid weight loss with unknown etiology, resulting in a decreased tolerance to chemotherapy, limited physical restoration, and complications. This phenomenon is more common in patients with ESCC compared to those with nongastrointestinal malignancies. Some preclinical findings suggested that the molecular cascades of cachexia pathogenesis widely impaired immune antitumor responses [48,49]. Several interventions to block the development of cachexia would invigorate antitumor immune functions [50,51]. On the other hand, leptin, an adipocyte-derived hormone increased in proportion to total adipose tissue mass, has been shown to involve in both innate and adaptive immune responses [52]. Recent studies found that it might contribute to the enhancement of the host immune response activated by PD-1/PD-L1 inhibitors in cancer patients [53]. It partially explained why patients with higher BMI would benefit from ICIs-based treatment.

The prognosis of ICIs-received ESCC was not only determined by the progression of disease itself, but also influenced by host-related factors. For unresectable ESCC in the immunotherapy era, a traditional TNM staging system was inadequate for directing the treatment protocols. The nomogram, a visual representation of a multiple regression analysis model, has been widely used in tumor prognostic analysis [54,55]. As a statistical tool, it could provide a tailored risk prediction, as well as compute individual predicted survival probabilities at different time points. This study’s nomogram was based on MLR, this inflammatory biomarker, while considering BMI and PS score. Tested through C-index, calibration curves and timeROC analysis, this composite nomogram exhibited better predictive accuracy than a single inflammatory indicator. Meanwhile, on the basis of the nomogram-generated score, we performed a risk stratification for patients, contributing to a better formulation of personalized regimens.

Here, we demonstrated two cases based on the calculation of the nomogram. Patient 1: BMI 18.25, PS score of 0, MLR 0.37, T4aN3M0; patient 2: BMI 20.96, PS score of 1, MLR 0.42, T4aN2M0 (Figure S3). Though they were both classified as stage IV, the nomogram calculated that they have different survival probabilities. The 24-month OS of the two patients were 50–60% and 20–30%, respectively. Since the nomogram incorporated routinely collected clinical factors, clinicians could use it during the initial consultation with patients and their families about prognosis and treatment advice. For instance, for patients with high scores, additional care and more frequent surveillance were recommended for earlier identification of disease progression and distant metastases [56]. As a result, we believe that our model has important clinical significance, as well as reference values to subsequent studies.

However, this study had some limitations. First, besides the small sample size, its retrospective, single-center design caused potential selection biases to be unavoidable. Second, the results of this study need further verifications in an external cohort. However, we validated the accuracy of the OS nomogram in our internal cohort by various methods including C-index, calibration curves, and timeROC curves. Third, since immunotherapy was made available in ESCC recently and partial patients had long-term survival, the median OS was not reached in our study, which necessitates a longer follow-up. Taken together, further researches are warranted to conduct continuously by seeking more data and collaborations prospectively for validating the model.

## 5. Conclusions

In conclusion, the findings of this study highlighted the prognostic significance of MLR in treatment-naive unresectable locally advanced or metastatic ESCC patients who had received immunotherapy combined with chemotherapy. Future studies should focus on the systemic inflammation and immune-activated response mediated by ICIs-based treatments. The nomogram based on the pretreatment MLR and clinical factors had better prognostic accuracy in individual risk prediction, which should be validated further in multicenter and prospective trials.

## Figures and Tables

**Table 1:** Clinical characteristics of patients at baseline.

Characteristics	Total (*n* = 81)	(%)
Gender		
Male	74	(91.36)
Female	7	(8.64)
Age (years)		
≤60	28	(34.57)
>60	53	(65.43)
Smoking history		
Yes	63	(77.78)
None	18	(22.22)
Drinking history		
Yes	58	(71.60)
None	23	(28.40)
BMI (kg/m²)		
≤18.5	19	(23.46)
>18.5	62	(76.54)
Location		
Cervical	9	(11.11)
Upper	8	(9.88)
Middle	39	(48.15)
Lower	25	(30.86)
Distant metastasis		
None	61	(75.31)
Yes	20	(24.69)
Liver metastasis		
None	74	(91.36)
Yes	7	(8.64)
Bone metastasis		
None	73	(90.12)
Yes	8	(9.88)
Lung metastasis		
None	75	(92.59)
Yes	6	(7.41)
TNM stage		
III	19	(23.46)
IV	62	(76.54)
PS score		
0	50	(61.73)
1	31	(38.27)
PD-1 or PD-L1		
PD-1 ICIs + chemotherapy	76	(93.83)
PD-L1 ICIs + chemotherapy	5	(6.17)
Chemotherapy		
Nab-PTX/PTX + platinum	57	(70.37)
5-FU + platinum	15	(18.52)
Other regimens	9	(11.11)
HB (g/L)		
≤120	20	(24.69)
>120	61	(75.31)
Neutrophil (×10^9^/L)		
Median (range)	4.45 (1.71–9.06)	
Lymphocyte (×10^9^/L)		
Median (range)	1.39 (0.45–3.23)	
Monocyte (×10^9^/L)		
Median (range)	0.54 (0.23–1.68)	
Platelet (×10^9^/L)		
Median (range)	260 (94–542)	

Abbreviations: BMI, body mass index; TNM, tumor-node-metastasis; PS score, performance status score; PD-1, programmed death-1; PD-L1, programmed death ligand-1; ICIs, immune checkpoint inhibitors; Nab-PTX, albumin-bound paclitaxel; PTX, paclitaxel; 5-FU, fluorouracil; HB, hemoglobin.

**Table 2:** Clinical variables of analyzed patients in different groups.

Variables	NLR ≤ 3.67	NLR > 3.67	*p*-Value ^a^	MLR ≤ 0.40	MLR > 0.40	*p*-Value ^a^
	(*n* = 50) (%)	(*n* = 31) (%)		(*n* = 45) (%)	(*n* = 36) (%)	
Gender			0.040			0.015
Male	43 (86.0)	31 (100.0)		38 (84.4)	36 (100.0)	
Female	7 (14.0)	0 (0.0)		7 (15.6)	0 (0.0)	
Age (years)			0.537			0.834
≤60	16 (32.0)	12 (38.7)		16 (35.6)	12 (33.3)	
>60	34 (68.0)	19 (61.3)		29 (64.4)	24 (66.7)	
Smoking history			0.625			0.591
Yes	38 (76.0)	25 (80.7)		34 (75.6)	29 (80.6)	
None	12 (24.0)	6 (19.4)		11 (24.4)	7 (19.4)	
Drinking history			0.155			0.544
Yes	33 (66.0)	25 (80.7)		31 (68.9)	27 (75.0)	
None	17 (34.0)	6 (19.4)		14 (31.1)	9 (25.0)	
BMI (kg/m²)			0.351			0.177
≤18.5	10 (20.0)	9 (29.0)		8 (17.8)	11 (30.6)	
>18.5	40 (80.0)	22 (71.0)		37 (82.2)	25 (69.4)	
Distant Metastasis			0.214			0.033
None	40 (80.0)	21 (67.7)		38 (84.4)	23 (63.9)	
Yes	10 (20.0)	10 (32.3)		7 (15.6)	13 (36.1)	
Liver Metastasis			0.100			0.041
None	48 (96.0)	26 (83.9)		44 (97.8)	30 (83.3)	
Yes	2 (4.0)	5 (16.1)		1 (2.2)	6 (16.7)	
Bone Metastasis			0.145			0.290
None	43 (86.0)	30 (96.8)		39 (86.7)	34 (94.4)	
Yes	7 (14.0)	1 (3.2)		6 (13.3)	2 (5.6)	
Lung Metastasis			0.196			0.083
None	48 (96.0)	27 (87.1)		44 (97.8)	31 (86.1)	
Yes	2 (4.0)	4 (12.9)		1 (2.2)	5 (13.9)	
TNM stage			0.220			0.019
III	14 (28.0)	5 (16.1)		15 (33.3)	4 (11.1)	
IV	36 (72.0)	26 (83.9)		30 (66.7)	32 (88.9)	
PS score			0.052			0.004
0	35 (70.0)	15 (48.4)		34 (75.6)	16 (44.4)	
1	15 (30.0)	16 (51.6)		11 (24.4)	20 (55.6)	
HB (g/L)			0.021			0.107
≤120	8 (16.0)	12 (38.7)		8 (17.8)	12 (33.3)	
>120	42 (84.0)	19 (61.3)		37 (82.2)	24 (66.7)	
PD-1 or PD-L1			1.000			0.375
PD-1 ICIs + chemotherapy	47 (94.0)	29 (93.5)		41 (91.1)	35 (97.2)	
PD-L1 ICIs + chemotherapy	3 (6.0)	2 (6.5)		4 (8.9)	1 (2.8)	
Chemotherapy			0.576			0.278
Nab-PTX/PTX + platinum	34 (68.0)	23 (74.2)		32 (71.1)	25 (69.4)	
5-FU + platinum	11 (22.0)	4 (12.9)		10 (22.2)	5 (13.9)	
Other regimens	5 (10.0)	4 (12.9)		3 (6.7)	6 (16.7)	

Abbreviations: NLR, neutrophil-to-lymphocyte ratio; MLR, monocyte-to-lymphocyte ratio; BMI, body mass index; TNM, tumor-node-metastasis; PS score, performance status score; HB, hemoglobin; PD-1, programmed death-1; PD-L1, programmed death ligand-1; ICIs, immune checkpoint inhibitors; Nab-PTX, albumin-bound paclitaxel; PTX, paclitaxel; 5-FU, fluorouracil. ^a^ *p* < 0.05 was recognized as statistical significance.

**Table 3:** Univariate and multivariate Cox regression analyses for PFS and OS.

		PFS						OS				
	Univariate Analysis			Multivariate Analysis			Univariate Analysis			Multivariate Analysis		
Variables	HR	95%CI	*p*-Value ^a^	HR	95%CI	*p*-Value ^a^	HR	95%CI	*p*-Value ^a^	HR	95%CI	*p*-Value ^a^
Gender												
Male		Reference						Reference				
Female	0.245	0.034–1.789	0.166				1.009	0.130–7.811	0.993			
Age (years)												
≤60		Reference						Reference				
>60	0.678	0.365–1.260	0.219				1.732	0.662–4.533	0.263			
BMI (kg/m²)												
≤18.5		Reference			Reference			Reference			Reference	
>18.5	0.537	0.276–1.041	0.066	0.572	0.293–1.119	0.103	0.383	0.156–0.939	0.036	0.323	0.128–0.818	0.017
Location												
Cervical		Reference						Reference				
Upper	0.922	0.200–4.253	0.917				1.273	0.253–6.410	0.770			
Middle	1.217	0.408–3.631	0.725				0.640	0.165–2.481	0.518			
Lower	2.740	0.915–8.201	0.072				1.154	0.294–4.523	0.837			
Metastasis												
None		Reference						Reference				
Yes	1.703	0.876–3.309	0.116				0.698	0.249–1.958	0.494			
TNM stage												
III		Reference			Reference			Reference				
IV	2.178	0.912–5.199	0.080	1.806	0.747–4.368	0.190	2.588	0.597–11.219	0.204			
PS score												
0		Reference						Reference			Reference	
1	1.256	0.663–2.379	0.485				2.493	1.016–6.119	0.046	1.718	0.687–4.301	0.247
HB (g/L)												
≤120		Reference						Reference				
>120	0.564	0.284–1.118	0.101				0.545	0.207–1.432	0.218			
MLR												
≤0.40		Reference			Reference			Reference			Reference	
>0.40	2.341	1.245–4.404	0.008	2.254	1.188–4.276	0.013	4.557	1.744–11.906	0.002	4.524	1.681–12.175	0.003

Abbreviations: PFS, progression free survival; OS, overall survival; HR, hazard ratio; CI, confidence interval; BMI, body mass index; TNM, tumor-node-metastasis; PS score, performance status score; HB, hemoglobin; MLR, monocyte-to-lymphocyte ratio. ^a^ *p* < 0.05 was recognized as statistical significance.

## Data Availability

Data supported the findings of the study could be acquired from the corresponding author by reasonable request.

## Supplementary Materials

The following supporting information can be downloaded at: https://www.mdpi.com/article/10.3390/curroncol29110702/s1. Figure S1: Kaplan–Meier curves of OS for ESCC patients in the chemotherapy cohort. Abbreviations: OS, overall survival; MLR, monocyte-to-lymphocyte ratio; ESCC, esophageal squamous cell carcinoma; Figure S2: The forest plot for OS and MLR level in different treatment groups. Abbreviations: HR, hazard ratio; OS, overall survival; MLR, monocyte-to-lymphocyte ratio; Figure S3: Two examples of OS prediction using the nomogram. Abbreviations: BMI, body mass index; MLR, monocyte-to-lymphocyte ratio; PS score, performance status score; OS, overall survival; TNM, tumor-node-metastasis; Table S1: Clinical characteristics of the immunotherapy plus chemotherapy cohort and the chemotherapy cohort. Abbreviations: BMI, body mass index; TNM, tumor-node-metastasis; PS score, performance status score; HB, hemoglobin; MLR, monocyte-to-lymphocyte ratio. ^a^ *p* < 0.05 was recognized as statistical significance.

## Abbreviations

ESCC	esophageal squamous cell carcinoma
MLR	monocyte-to-lymphocyte ratio
PD-1	programmed death-1
PD-L1	programmed death ligand-1
OS	overall survival
PFS	progression free survival
C-index	concordance index
timeROC	time-dependent receiver operating characteristic
PS score	performance status score
BMI	body mass index
AUC	area under ROC curve
TNM	tumor-node-metastasis
ICI	immune checkpoint inhibitor
TMB	tumor mutational burden
ctDNA	circulating tumor DNA
NLR	neutrophil-to-lymphocyte ratio
NSCLC	non-small-cell lung cancer
GC	gastric cancer
AJCC	American Joint Committee on Cancer
ECOG	Eastern Cooperative Oncology Group
HB	hemoglobin
Nab-PTX	albumin-bound paclitaxel
PTX	paclitaxel
5-FU	fluorouracil
CR	complete response
PR	partial response
SD	stable disease
PD	progressive disease
ORR	objective remission rate
DCR	disease control rate
HR	hazard ratio
CI	confidential interval
CRC	colorectal cancer
TTP	time to progression
HCC	hepatocellular carcinoma
TILs	tumor-infiltrating lymphocytes
TAMs	tumor-associated macrophages
