# Supplementary material for: Nomogram Based on Monocyte-to-Lymphocyte Ratio to Predict Survival of Unresectable Esophageal Squamous Cell Carcinoma Who Receive First-Line PD-1/PD-L1 Inhibitors Combined with Chemotherapy
Source: Curr Oncol. 2022 Nov 18;29(11):8937–54. doi: 10.3390/curroncol29110702 (PMC9689525; doi:10.3390/curroncol29110702)
Supplement: Supplementary file 1 [file curroncol-29-00702-s001.zip › curroncol-1973149-supplementary.pdf]

Supplementary materials:

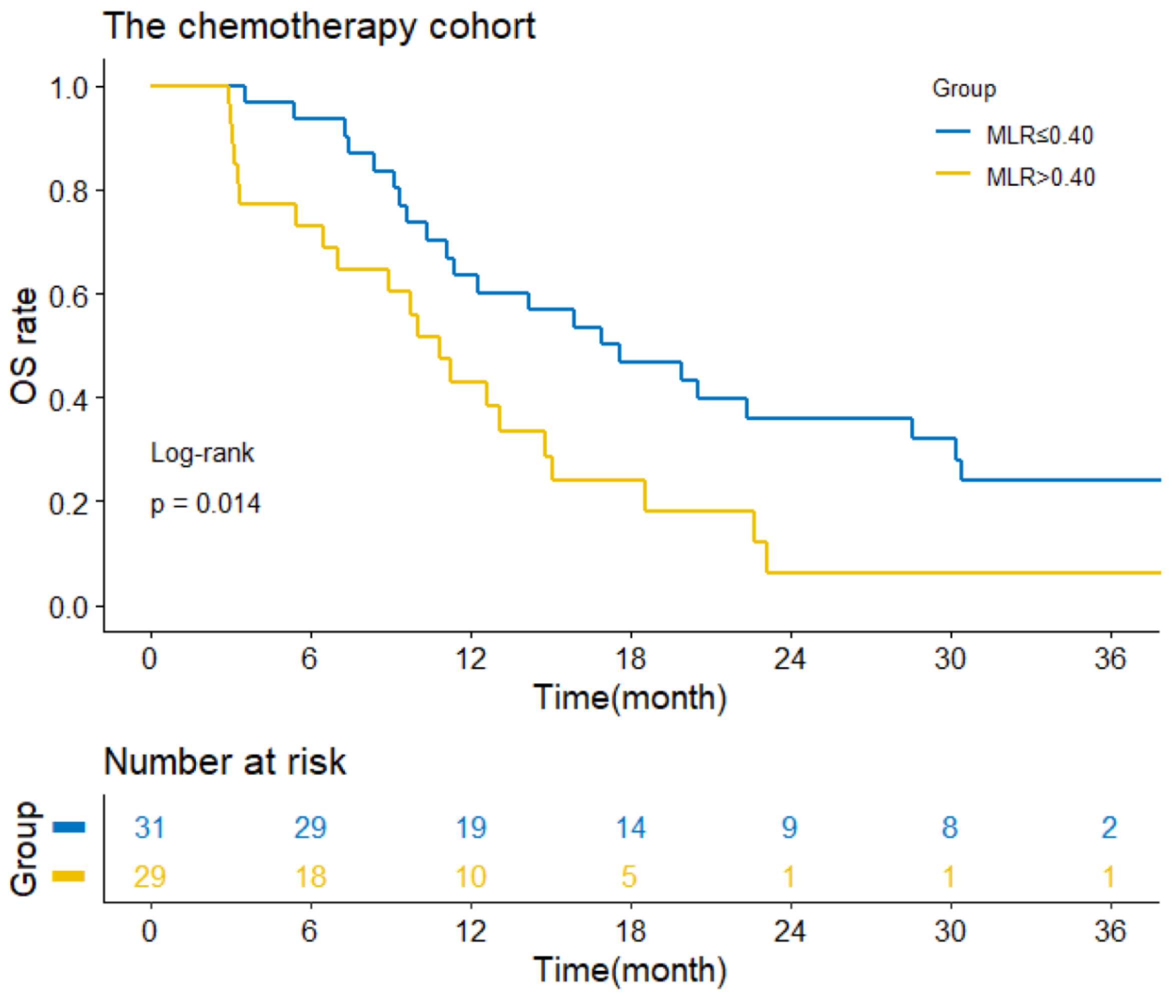

Figure S1: Kaplan–Meier curves of OS for ESCC patients in the chemotherapy cohort. Abbreviations: OS, overall survival; MLR, monocyte-to-lymphocyte ratio; ESCC, esophageal squamous cell carcinoma.

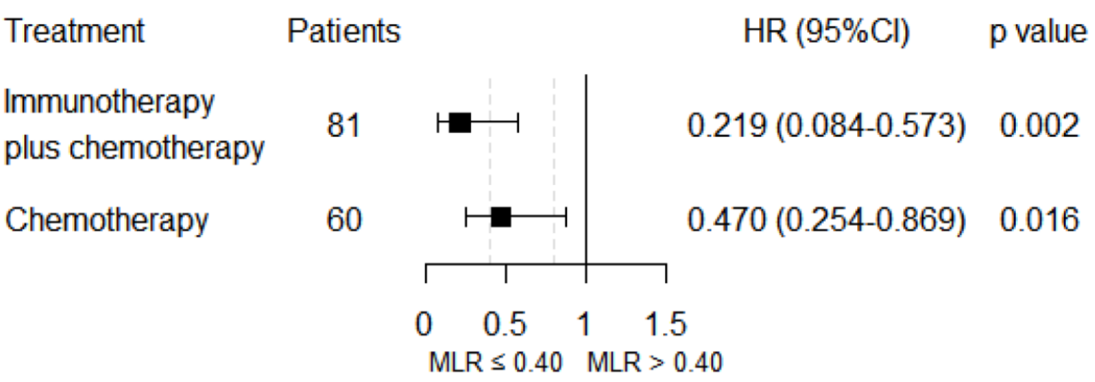

Figure S2: The forest plot for OS and MLR level in different treatment groups. Abbreviations: HR, hazard ratio; OS, overall survival; MLR, monocyte-to-lymphocyte ratio.

Variable	Points
BMI ≤ 18.5kg/m²	7.48
MLR > 0.40	10
PS score = 1	3.59

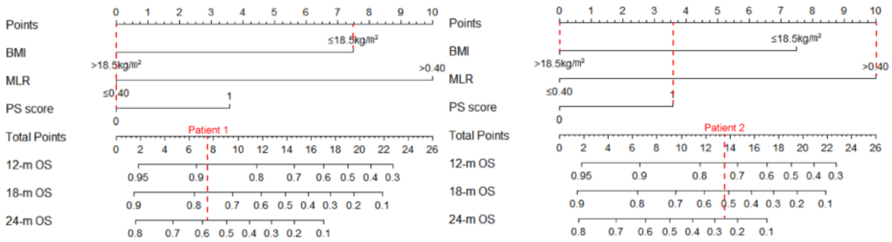

	Gender	Age	BMI	Location	Distant metastasis	TNM stage	PS score	MLR	Points	12-m OS	18-m OS	24-m OS
Patient 1	Male	65	18.25	Lower	None	T4aN3M0	0	0.37	7.48	80-90%	70-80%	50-60%
Patient 2	Male	66	20.96	Middle	None	T4aN2M0	1	0.42	13.59	70-80%	50-60%	20-30%

Figure S3: Two examples of OS prediction using the nomogram. Abbreviations: BMI, body mass index; MLR, monocyte-to-lymphocyte ratio; PS score, performance status score; OS, overall survival; TNM, tumor-node-metastasis.

Table S1 Clinical characteristics of the immunotherapy plus chemotherapy cohort and the chemotherapy cohort.

**Characteristics**	**Immunotherapy + chemotherapy n=81 (%)**	**Chemotherapy n=60 (%)**	***p* value [a]**
**Gender**			0.372
Male	74 (91.36%)	52 (86.67%)	
Female	7 (8.64%)	8 (13.33%)	
**Age (years)**			0.509
≤60	28 (34.57%)	24 (40.00%)	
>60	53 (65.43%)	36 (60.00%)	
**BMI (kg/m²)**			0.213
≤18.5	19 (23.46%)	9 (15.00%)	
>18.5	62 (76.54%)	51 (85.00%)	
**Location**			0.289
Cervical	9 (11.11%)	4 (6.67%)	
Upper	8 (9.88%)	7 (11.67%)	
Middle	39 (48.15%)	22 (36.67%)	
Lower	25 (30.86%)	27 (45.00%)	
**Distant metastasis**			0.790
None	61 (75.31%)	44 (73.33%)	
Yes	20 (24.69%)	16 (26.67%)	
**TNM stage**			0.624
III	19 (23.46%)	12 (20.00%)	
IV	62 (76.54%)	48 (80.00%)	
**PS score**			0.846
0	50 (61.73%)	38 (63.33%)	
1	31 (38.27%)	22 (36.67%)	
**HB (g/L)**			0.852
≤120	20 (24.69%)	14 (23.33%)	
>120	61 (75.31%)	46 (76.67%)	

**MLR**			0.647
MLR≤0.40	45 (55.56%)	31 (51.67%)	
MLR>0.40	36 (44.44%)	29 (48.33%)	

Abbreviations: BMI, body mass index; TNM, tumor-node-metastasis; PS score, performance status score; HB, hemoglobin; MLR, monocyte-to-lymphocyte ratio. [a] $p < 0.05$ was recognized as statistical significance.
